# Sim-to-real domain adaptation based completion level recognition for autonomous micro-drilling in biomedical application

**DOI:** 10.1038/s41598-025-26600-1

**Published:** 2025-11-27

**Authors:** Enduo Zhao, Saul Alexis Heredia Perez, Kanako Harada

**Affiliations:** 1 Graduate School of Engineering, The University of Tokyo, Tokyo, 113-8654 Japan; 2 Center for Disease Biology and Integrative Medicine, Graduate School of Medicine, The University of Tokyo, Tokyo, 113-8654 Japan; 3 Present Address: School of Biomedical Engineering, Tsinghua University, Beijing, 100084 China

**Keywords:** Sim-to-real transfer, Domain adaptation, Autonomous micro-drilling, Biomedical application, Biological techniques, Computational biology and bioinformatics, Engineering, Mathematics and computing, Medical research

## Abstract

Micron-level precision in drilling thin bone structures is essential in both surgical and neuroscience applications but remains technically challenging due to tissue fragility and anatomical variability. Traditional manual methods are slow and error-prone, while systems based on preoperative imaging lack adaptability to intraoperative changes. We previously proposed a convolutional neural network based autonomous micro-drilling system, enabling real-time control without prior bone knowledge. However, its reliance on manual annotation limited scalability and accuracy. In this study, we enhance the system through a sim-to-real domain adaptation model using synthetic data generated from a photorealistic simulator. The novelty of this work is a task-specific adversarial model that bridges the domain gap, significantly reducing annotation time (from 600 s/frame to 1.8 s/frame) while achieving a success rate of 85% (up from 80%) in 20 trials of eggshell drilling. These results demonstrate the feasibility and effectiveness of simulation-based training and domain adaptation for improving autonomous micro-drilling performance in biomedical applications.

## Introduction

In surgical procedures involving drilling or milling of very thin bone structures, such as endoscopic endonasal approaches of the skull base^1,2^ or percutaneous cochlear implantation^3^, achieving micron-scale precision presents significant challenges due to the small, non-uniform bone thickness and individual anatomical variability. These procedures are typically performed manually with handheld drills, which are time-consuming, skill-intensive, and prone to inconsistency. Although computed tomography (CT)-based navigation systems assist in surgery^4^, accuracy often remains limited to 1–2 mm, which is inadequate near critical structures such as the cochlea or optic nerve^2^.

Similarly, in biomedical research involving rodents, precise and minimally drilling on the cranial bone, which can be as thin as 300 ${\upmu }$µm^5^, are extremely demanding and essential for a wide range of in vivo neuroscience experiments, such as neural recording^6,7^ or optical imaging^8,9^. However, variability in bone thickness across strains and ages makes manual drilling unsafe and imprecise^10^. Robotic systems using preoperative imaging such as micro-CT^11^ or optical coherence tomography (OCT)^12,13^ have been explored, but they struggle to adapt to intraoperative changes and still depend heavily on human intervention.

To address these challenges, Marinho et al.^14^ demonstrated the feasibility of autonomous micro-drilling using a robotic manipulator equipped with a micro-drill, while Zhao et al.^15^ proposed a CNN-based method that recognizes pixel-wise drilling completion level from 2D images, generating completion level maps that guide real-time trajectory adjustment, allowing adaptation to anatomical variability and enabling safe, fully autonomous micro-drilling without prior knowledge of bone shape or thickness. The system was validated initially on chicken eggs, whose shells exhibit structural similarities with bone tissue^16^, achieving a success rate of 80% with an average drilling time of 16.8 min, demonstrating its feasibility. However, the method relies on a well-labeled dataset with ground truths of pixel-wise completion maps ranging from 0% to 100%. Since manual annotation at this level is impractical, images were labeled into five discrete levels (0%, 25%, 50%, 75%, 100%) and smoothed with a Gaussian filter to approximate continuous maps. Despite simplification, annotating 518 images still required around 90 hours (600 s/frame). Furthermore, the smoothing introduced noise, limiting accuracy and contributing to potential failure.

In order to address this issue, Heredia et al.^17^ developed a simulation platform for micro-drilling leveraging Isaac Sim simulator to generate synthetic data. It is expected that photorealistic synthetic images with ground truths can be created by the simulator automatically, reducing annotation effort and improving the accuracy of autonomous micro-drilling, which inevitably runs into another challenge of the domain gap. In the context of this work, the domain gap is considered as the difference in the performance of the model trained with synthetic dataset when it is evaluated against real images.

Bridging the domain gap remains a challenge, with common approaches including domain randomization (DR) and domain adaptation (DA). DR improves generalization by introducing randomized variations in synthetic images (e.g., lighting, texture or background^18–20^), without requiring photorealism^20^, but its effectiveness depends on the relevance of variations and may suffer from reduced realism. In contrast, DA learns domain-invariant features through end-to-end training^21–23^, often using adversarial learning^24^ or style transfer techniques^25^, and has shown success in cross-domain tasks such as semantic segmentation.

In our task of completion level recognition, the target domain (real images) is fixed and abundant unlabeled data can be collected, while high realism and semantic fidelity are essential. Therefore, DA is more suitable than domain randomization, and our task naturally falls into the category of unsupervised domain adaptation (UDA). However, conventional UDA methods primarily focus on aligning feature distributions and do not directly enforce pixel-level consistency^21–23^, which is problematic for our application because subtle textures such as the membrane exposed after penetrating the eggshell are critical cues for reliable recognition.

To overcome this limitation, we adopt a style transfer-based UDA approach that transforms synthetic images into the style of real images via adversarial training in a generative adversarial network (GAN) framework, leveraging prior experience from real-image-based eggshell drilling^15^. While models like CycleGAN^26^ can generate realistic images, they lack task-awareness. CyCADA^25^, combining pixel-level and feature-level alignment, enabling improved task-specific adaptation and preserving fine-grained texture details crucial for our application.

In this work, a synthetic dataset is generated using the existing micro-drilling simulator as the foundation for training. Building on this, a task-specific sim-to-real DA model is proposed based on the structure of CyCADA, with an adapted output structure and a loss function customized to the completion level recognition task, which represents the main contribution of this study. The proposed model is evaluated against state-of-the-art methods and validated through autonomous eggshell drilling experiments using the same robotic system in previous research^15^. Preliminary results demonstrate the feasibility of the model, significantly reducing annotation effort while improving success rate in robotic micro-drilling for biomedical applications.

## Synthetic data creation with micro-drilling simulator

### Micro-drilling simulator

**Figure 1:**
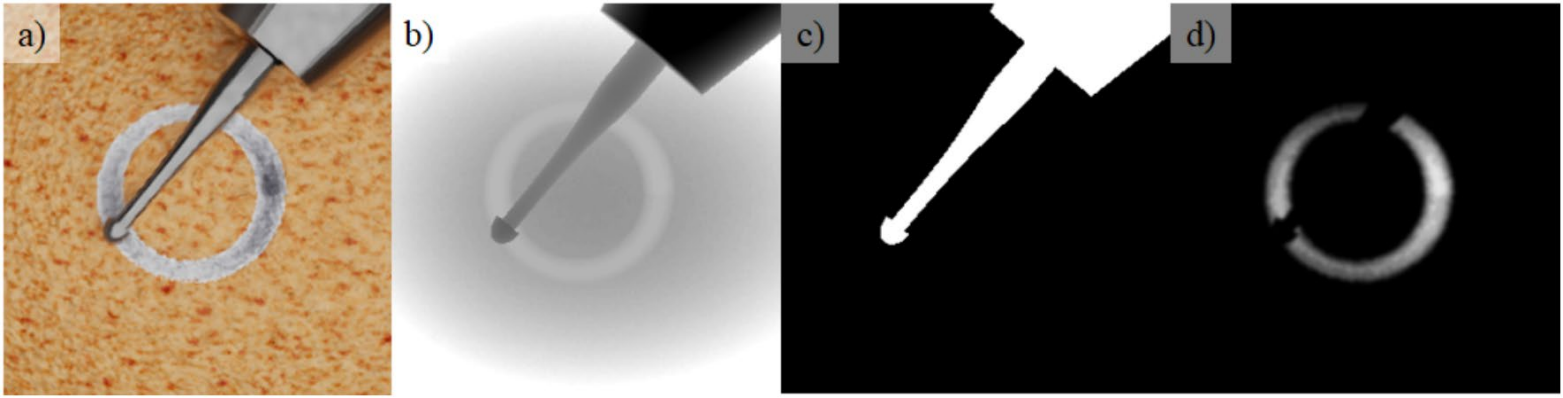
A simulation frame showing (**a**) RGB image, (**b**) depth map, (**c**) segmentation of drill, and (**d**) completion level map.

The micro-drilling simulator developed by Heredia et al.^17^ is utilized to generate synthetic data for the study. The simulator integrates a Digital Twin corresponding to the robotic platform used in drilling experiments, enabling simulation of the drilling process on bone tissue. To maintain consistency with previous studies, an egg was chosen as the target object for drilling. The simulator provides an accurate and interactive simulation of the drilling procedure, featuring realistic haptic and auditory feedback. The simulator is operated via the Touch X haptic device (3D Systems, USA), which replicates the operation of the physical robotic system, ensuring that the simulation closely mirrors real-world robot interactions. Additionally, the simulator leverages Isaac Sim (NVIDIA, USA) for real-time ray tracing, producing photorealistic images that replicate the drilling process (see Fig. 1a), including changes in shape and color of the eggshell during the procedure. Furthermore, the simulator can output depth maps and semantic segmentation information of drill (see Fig. 1b and c), making it a powerful tool for generating diverse synthetic datasets to train machine learning models. For this work, the simulator is modified to include the capability to control the motion of the drill programmatically, enabling the generation of precise circular drilling trajectories, as performed by the robot during autonomous micro-drilling.

### Synthetic data generation

Synthetic data are collected in the simulator by programmatically generating circular drilling trajectories. To introduce variability, the starting position of the drilling trajectory is adjusted randomly, producing synthetic data with different drilling offsets. When the drilling trajectory is not perfectly centered, the resulting drilling area exhibit variations in depth. The simulator, in its original form, does not include a built-in function to produce completion level maps for the drilling process. These maps are generated as a postprocessing step, shown in Fig. 1d. First, the simulator records color, depth, and segmentation mask images during operation. The drilling completion level for each pixel (*x*, *y*) in each frame *i* is then calculated by Eq. (1), where *clevel* is the completion level map, *depth* is the depth map, $depth_0$ is the initial depth map before drilling starts, and $thickness=0.5$ (mm) is the eggshell thickness in the simulator. This results in grayscale maps where black (0%) represents areas not drilled or occluded by the drill, and white (100%) indicates fully drilled regions. Unlike manual annotation, which provides only a few discrete levels (see Section ), this approach generates maps with continuous drilling completion levels, offering a more detailed and precise representation of the drilling process. The average processing time for each frame is $1.8 \pm 0.7$ s, much shorter than manual annotation (about 600 s).

1 $$clevel_{i} (x,y) = clamp\left( {\frac{{(depth_{i} (x,y) - depth_{0} (x,y))}}{{thickness}},0,1} \right)$$

Using this method, a synthetic dataset was generated with a total of 6,286 data, containing RGB images, segmentation of drill and completion level maps.

## Sim-to-real domain adaptation model

**Figure 2:**
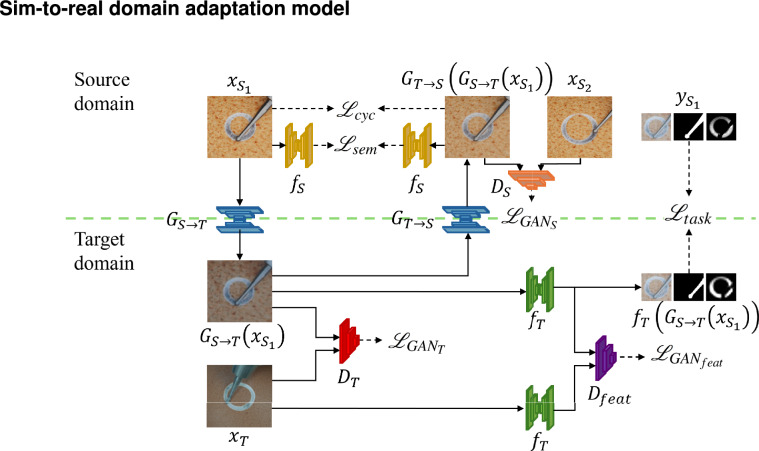
Overview of the proposed model. Two task nets separately for source domain and target domain ($f_S$ and $f_T$), an pixel-level GAN from source domain to target domain (generator $G_{S \rightarrow T}$ and discriminator $D_T$), an pixel-level GAN from target domain to source domain (generator $G_{T \rightarrow S}$ and discriminator $D_S$), a feature level GAN (discriminator $D_{feat}$) are shown. $x_{S_1}$ and $x_{S_2}$ are two synthetic images in source domain $X_{S}$, and $y_{S_1}$ is the ground truth of $x_{S_1}$. $x_T$ is a real image in target domain $X_T$. Besides, source recognition $f_T(G_{S \rightarrow T}(X_{s_1}$)), source image stylized as target $G_{S \rightarrow T}(X_{s_1}$) and reconstructed source image $G_{T \rightarrow S}(G_{S \rightarrow T}(X_{s_1}$)) are served as the input and output of those networks. It is also depicted the components of the overall loss $\mathscr {L}_{all}$: the task loss $\mathscr {L}_{task}$, the source and target pixel-level GAN losses $\mathscr {L}_{GAN_S}$ and $\mathscr {L}_{GAN_T}$, the feature level GAN loss $\mathscr {L}_{GAN_{feat}}$, the source and target semantic consistency losses $\mathscr {L}_{sem}$, and the source cycle loss $\mathscr {L}_{cyc}$.

The proposed domain adaptation model for sim-to-real completion level recognition uses the same pixel-level GANs and feature-level GAN of the CyCADA^25^, and modifies the task nets $f_S$ and $f_T$ to output the completion level recognition results rather than the segmentation results. The according loss functions are changed due to the change of task. It is discussed the overview, loss functions and individual components of the network architecture in detail in the following sections.

### Overview and loss function

The overview of the model is shown in Fig. 2. The problem of UDA is considered, where synthetic images in source domain $X_{S}$, ground truths in source domain $Y_{S}$ , and real images in target domain $X_{T}$ are provided, but no ground truth in the target domain. $x_{S_1}$, $x_{S_2}$, $x_T$ are sample images in these domains and $y_{S_1}$ is the ground truth of $x_{S_1}$. The goal is to learn a task net model $f_T$ that can correctly recognize the completion level for the real images $X_{T}$.

First, a CNN model $f_S$ is needed to pre-train which can recognize the completion level on the synthetic images in source domain. However, $f_S$ can not be expected to preform well on the real images in target domain because of the domain gap. Therefore, the goal is to train another CNN $f_T$ based on $f_S$ for the recognition on real images in target domain. To mitigate the effects of domain gap, adversarial adaptation approach is applied which learns to map samples across domains such that an adversarial discriminator is unable to distinguish the domains. By mapping samples into a common space, the proposed model is able to learn from the source data while still generalizing well to the target data.

To achieve this, a pixel-level GAN is introduced including a generator from the source to the target $G_{S \rightarrow T}$ to generate target samples from source domain and an adversarial discriminator $D_T$ to distinguish between real target images and source-target images. This process is associated with the loss function $\mathscr {L}_{GAN_T}$. This objective ensures that $G_{S \rightarrow T}$ generates convincing target samples from source images, enabling the learning of a target model $f_T$ by minimizing the task loss function $\mathscr {L}_{task}$.

However, the GAN loss $\mathscr {L}_{GAN_T}$ cannot ensure that $G_{S \rightarrow T}(x_{S_1})$ preserves the structure or content of the original sample $x_{S_1}$. To address this issue, another GAN is introduced whose generator $G_{T \rightarrow S}$ is from target to source and trained according to the same GAN loss $\mathscr {L}_{GAN_S}$. This setup enforces cycle-consistency by requiring that a sample mapped from source to target and back to source reproduces the original sample, that is, $(G_{T \rightarrow S}(G_{S \rightarrow T}(x_{S_1})) \approx x_{S_1}$ and $(G_{S \rightarrow T}(G_{T \rightarrow S}(x_T)) \approx x_T$. The cycle-consistency is enforced by adding an L1 penalty on the reconstruction error, known as the cycle-consistency loss $\mathscr {L}_{cyc}$.

Furthermore, by leveraging source ground truth, it is also enforced high semantic consistency before and after image translation. The pre-trained source task model $f_S$ is fixed and used as a noisy labeler to ensure that an image is classified similarly by this model both before and after translation. This approach allows to compute the semantic consistency loss $\mathscr {L}_{sem}$ before and after image translation.

In addition to the pixel-level adaptation method that integrates cycle consistency, semantic consistency, and adversarial objectives, a feature-level GAN is also proposed. This GAN discriminates between the features or semantics of two image sets as processed by a task network, introducing an additional feature-level GAN loss $\mathscr {L}_{GAN_{feat}}$.

Taken together, these loss functions constitute our complete objective ${L}_{all}$:

2 $$\begin{aligned} \begin{aligned} \mathscr {L}_{all} = \mathscr {L}_{task} + \mathscr {L}_{GAN_S} + \mathscr {L}_{GAN_T} +\mathscr {L}_{GAN_{feat}} +\mathscr {L}_{sem} + \mathscr {L}_{cyc}, \end{aligned} \end{aligned}$$

where $\mathscr {L}_{GAN_S}$, $\mathscr {L}_{GAN_T}$, $\mathscr {L}_{GAN_{feat}}$, $\mathscr {L}_{sem}$, $\mathscr {L}_{cyc}$ and their corresponding weights (omitted in the equation) are consistent with those used in the CyCADA model for the GTA5-to-Cityscapes semantic segmentation task^25^. Regarding the weight of $\mathscr {L}_{task}$, although it differs from the original CyCADA task loss (discussed in detail below), we performed sensitivity analysis around its original weight in CyCADA ($\lambda =1$) and found the effect to be minimal (available in Supplementary Table S2). Therefore, the weight of $\mathscr {L}_{task}$ is set to 1.

We now turn to $\mathscr {L}_{task}$. Unlike the semantic segmentation task used in CyCADA, our completion level recognition task comprises two sub-tasks: drilling area detection and completion prediction. So the ground truth $y_{S_1}$ or the recognition result $f_S(x_{S_1})$ will have three channels. The first channel ($y_{S_1}[1]$ and $f_S(x_{S_1})[1]$) is the drilling area detection result that is described as a 4-dimensional vector ($x_{1}$, $y_{1}$, $x_{2}$, $y_{2}$) to represent the coordinates of the upper left and lower right corners of the bounding box. The other two channels are for completion prediction, in which the second channel ($y_{S_1}[2]$ or $f_S(x_{S_1})[2]$) is a binary image of drill segmentation while the third channel ($y_{S_1}[3]$ or $f_S(x_{S_1})[3]$) is a grayscale image of completion level map.

As a result, the task loss $\mathscr {L}_{task}$ is the sum of the loss function of bounding box detection $\mathscr {L}_{bb}$ for the drilling area, and the loss function of the completion prediction $\mathscr {L}_{com}$. And $\mathscr {L}_{bb}$ is the sum of the localization loss $\mathscr {L}_{loc}$ and the confidence loss $\mathscr {L}_{conf}$, while $\mathscr {L}_{com}$ is the sum of the drill segmentation loss $\mathscr {L}_{drill}$ and completion level grayscale image loss $\mathscr {L}_{gray}$. That is

3 $$\begin{aligned} \begin{aligned} \mathscr {L}_{task}&= \mathbb {E}_{(x_{S_1}, y_{S_1}) \sim {(X_S, Y_S)}}[\frac{1}{N} \mathscr {L}_{bb} + \mathscr {L}_{com}] \\&= \mathbb {E}_{(x_{S_1}, y_{S_1}) \sim {(X_S, Y_S)}}[\frac{1}{N}( \mathscr {L}_{loc} + \mathscr {L}_{conf}) + \mathscr {L}_{drill} + \mathscr {L}_{gray}] \end{aligned} \end{aligned}$$

where $\mathbb {E}$ denotes the expectation, representing the average value of the loss function over all possible samples $(x_{S_1}, y_{S_1})$ randomly drawn from the source domain $(X_S, Y_S)$. *N* refers to the number of matched default boxes. If $N = 0$, we set the loss to 0. For $\mathscr {L}_{bb}$, $\mathscr {L}_{loc}$ is a Smooth L1 loss between the predicted box and the ground truth box and $\mathscr {L}_{conf}$ is the softmax loss over *N* multiple classes confidence. On the other hand, for $\mathscr {L}_{com}$, $\mathscr {L}_{drill}$ is For 1-way classification with a cross-entropy loss and $\mathscr {L}_{gray}$ is an L2 loss between the predicted completion level and the ground truth:

$$\begin{aligned}&\mathscr {L}_{loc} = \sum _{n}^N \sum _{i \in \{x_1, y_1, x_2, y_2\}} smooth_{L_1}(f_T(G_{S \rightarrow T}(x_{S_1}))[1]_n^{(i)}-y_{S_1}[1]_n^{(i)}), \\&\mathscr {L}_{conf} = - \sum _{n}^N y_{S_1}[1]_n \cdot \log \sigma (f_T^{(n)}(G_{S \rightarrow T}(x_{S_1}))[1]_n), \\&\mathscr {L}_{drill} = - f_T (G_{S \rightarrow T}(x_{S_1}))[2] \cdot \log y_{S_1}[2] - (1-f_T(G_{S \rightarrow T}(x_{S_1}))[2]) \cdot \log (1- y_{S_1}[2]), \\&\mathscr {L}_{com} = ||(f_T(G_{S \rightarrow T}(x_{S_1}))[3]-y_{S_1}[3]||_2. \end{aligned}$$

where $\sigma$ in $\mathscr {L}_{conf}$ denotes the softmax function.

Combining the above, the complete loss function ${L}_{all}$ can be calculated by equation (2) and a target model $f_T$ can be solved according to the optimization problem:

4 $$\begin{aligned} \begin{aligned} f_T^* = \mathop {\arg \min }\limits _{f_T} \mathop {\mathop {\min }\limits _{G_{S \rightarrow T}}}\limits _{G_{T \rightarrow S}} \mathop {\max }\limits _{D_S, D_T} \mathscr {L}_{all} \end{aligned} \end{aligned}$$

### Network architecture

The network architectures of both generator and discriminator in pixel-level GANs $G_{S \rightarrow T}$, $D_T$, $G_{T \rightarrow S}$ and $D_S$ are the same as CyCADA^25^. Since the task of our model is different, the task net *f* (both $f_S$ and $f_T$) change a lot as well as the discriminator of the feature-level GAN $D_{feat}$. The architecture of each net will be briefly summarized in the following sections, and more details are available in the supplementary document.

The architectures of task nets $f_S$ and $f_T$ are modified from our previous completion level recognition CNN^15^ (shown in Supplementary Fig. S1a). ResNet-50^27^ is used as the feature extractor, followed by convolutional and deconvolutional layers with ResSkip blocks for multi-scale feature fusion. The network has two output branches: one for drilling area detection, and the other for completion prediction which has a size of 128$\times$128$\times$2 representing drill segmentation and completion level map.

The discriminator of the feature level GAN $D_{feat}$ has a dual-stream architecture that processes the bounding box and the grayscale maps using convolutional and fully connected layers, respectively. The architecture is shown in Supplementary Fig. S1b. The extracted features are concatenated and passed through a softmax classifier to predict whether the input comes from the source or target domain.

The pixel-level GAN consists of a generator ($G_{S \rightarrow T}$ or $G_{T \rightarrow S}$) with residual blocks for domain translation and a discriminator ($D_T$ or $D_S$) with convolutional layers for pixel-wise real/fake classification.

## Model training and evaluation

### Training dataset preparation

Model training requires two datasets: a synthetic dataset with images and ground truths, and a real dataset with only images.

Through synthetic data generation described earlier, we gathered 6,286 synthetic data from the eggshell drilling simulator, containing RGB images, segmentation of drill and completion level maps. The ground truth bounding box coordinates ($x_{1}$, $y_{1}$, $x_{2}$, $y_{2}$) are derived from the minimum and maximum pixel positions of non-zero values in the grayscale maps. Since manually annotated bounding boxes in the real dataset typically include a small margin to avoid edge errors, we compared them with the coordinates calculated from the grayscale maps and found an average difference of about 10 pixels. This difference was used as compensation: 10 pixels were subtracted from $x_{1}$ and $y_{1}$ and added to $x_{2}$ and $y_{2}$.

On the other hand, 518 real images were collected from manual eggshell drilling experiments that comprised the real dataset, as detailed in our previous study^15^.

As the training procedure is set as unsupervised since ground truth for real dataset are not provided, validation step is not necessary so all data in synthetic dataset and 460 samples in real dataset are applied in training step. The rest 58 real images are used for evaluation and showing the performance of the trained model.

### Training procedure

In our proposed model, a task net $f_S$ that can recognize the completion level on the synthetic image in source domain need to be pre-trained. The training details of the task net and the whole model are available in Supplementary Table S3 and S4.

### Evaluation

Although in the training phase, only the ground truths of the synthetic images in the source domain are provided, the ground truths of the real images are actually present. These ground truths are created manually, and the annotation methods and processes are detailed in the previous study^15^. Consequently, it is possible to evaluate the performance of the proposed $f_T$ using 58 real images in the target domain.

As metrics, mean average precision (mAP) is used to evaluate the performance of the drilling area detection sub-task, while for the completion prediction sub-task, Mean Intersection over Union (mIoU) and Mean Absolute Percentage Error (MAPE) are used to evaluate the results of drill segmentation and completion level map, respectively. We compare the performance of the target domain task net $f_T$, which is the goal of the training, and the source domain task net $f_S$, which has only been pre-trained on synthetic data, both on real dataset. Furthermore, it was already counted with a CNN model (denoted $f_R$) trained solely on real images and ground truths, described in the previous study^15^ which was also compared.

**Figure 3:**
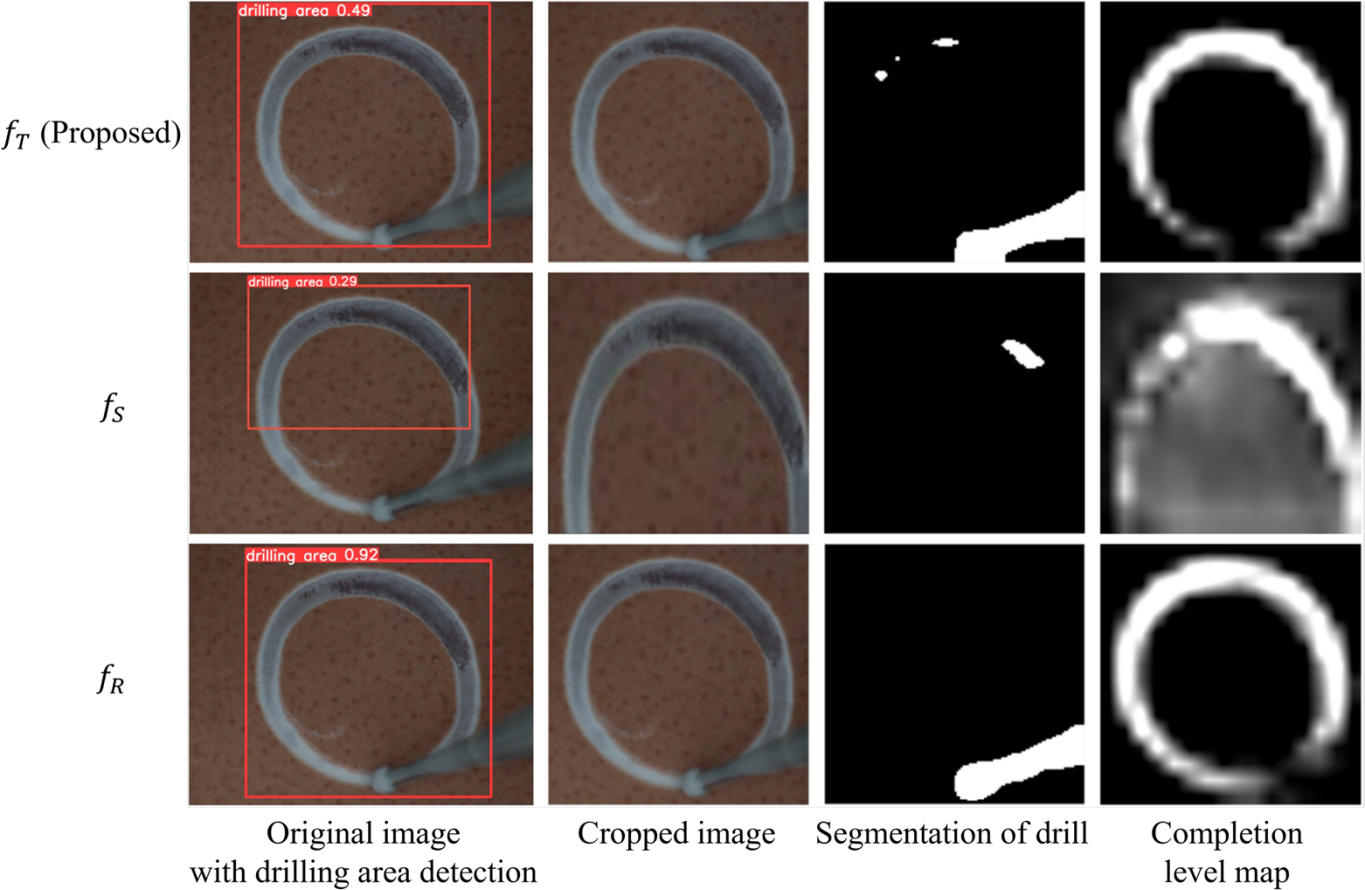
Visualization of the completion level recognition results from $f_T$, $f_S$ and $f_R$ on a real eggshell drilling image.

The evaluation result is shown in Table 1. $f_T$ can reach up 69.3 of mAP and 22.75% of MAPE. From the result, the performance of $f_T$ is significantly improved compared to $f_S$ (31.7 of mAP and 88.90% of MAPE) while approaching $f_R$ (78.5 of mAP and 15.05% of MAPE), which is inline with our expectation. To present the results more intuitively, the performance of model $f_T$, $f_S$ and $f_R$ on a real image sample are separately shown in Fig. 3. Additionally, the inference speed of model $f_T$ is 72 Hz, meeting the requirement of real-time.

Although the performance of proposed $f_T$ still does not reach that of $f_R$, the gap is acceptable considering the significant difference in time required for dataset acquisition (1.8 s/frame vs. 600 s/frame). Furthermore, since all models were evaluated using manually annotated ground truths, which inherently contain unavoidable errors and noise, these results can only serve as a preliminary reference. The performance of proposed $f_T$ must ultimately be verified by autonomous robotic drilling experiments.

**Table 1:** Performance evaluation of $f_T$, $f_S$, $f_R$, and comparison with other methods.

Model	mAP	mIoU (%)	MAPE (%)
$f_T$ [Proposed]	69.3	80.9	22.8
$f_S$ [Non-adaptive transfer learning]	31.7	20.4	88.9
$f_R$	78.5	85.1	15.1
Augmentation-based method	33.9	20.7	84.5
AdaptSegNet^28^	45.7	47.5	62.2
SPGAN-DA^29^	72.1	77.4	17.6
FADA^30^	$\hbox {N.A.}^{1}$	64.2	28.4
PyCDA^31^	$\hbox {N.A.}^{1}$	71.0	24.1

$^{1}$N.A. indicates the model failed to obtain meaningful results in the sub-task of drilling area detection.

### Comparison with other methods

Table 1 also presents a comparison of $f_T$ with non-adaptive transfer learning, augmentation-based methods, and several state-of-the-art DA methods^28–31^. Non-adaptive transfer learning refers to directly training on synthetic dataset in source domain without any modification, which corresponds to $f_S$ in our model. Augmentation-based methods apply various enhancement techniques to the synthetic dataset, and the method we used for comparison includes blur and noise augmentation, color adjustment, geometric transformations, and resolution alterations. It is worth noting that some of these methods were not architecturally designed for detection and therefore fail to provide acceptable input for the subsequent task. For these methods, the input images were manually cropped, and only their performance on the prediction task was evaluated.

The comparison results show that non-adaptive transfer learning and augmentation-based methods perform significantly worse. This demonstrates that simple transfer learning or data augmentation cannot effectively bridge the substantial domain gap between synthetic and real images, highlighting the necessity of DA approach. Among the DA methods, FADA and PyCDA performed poorly potentially because they don’t use GANs, relying instead on class-prototype alignment and cycle-consistency loss, respectively. Thus they fail to directly address the significant pixel-level differences. AdaptSegNet, which does use a GAN, showed some improvement but its focus on feature alignment still falls short of handling the critical pixel-level semantic information needed for our task. This explains why both SPGAN-DA and our method, which leverage GANs for cross-domain alignment, performed better on the eggshell images.

Nevertheless, our method still showed potential for outperforming SPGAN-DA. SPGAN-DA’s single end-to-end network tightly couples pixel and feature adaptation, which could be a bottleneck in complex scenarios such as rodent skull images. In contrast, our method performs adaptation at both the pixel and feature levels simultaneously, decoupling the image style transfer and feature alignment steps to allow for independent optimization. This design provides stronger robustness and adaptability, which is crucial for our ultimate goal of micro-drilling on bone or other biomedical tissues.

## Experiment

In this section, an autonomous eggshell drilling experiment is conducted to validate the performance of our proposed model $f_T$.

**Figure 4:**
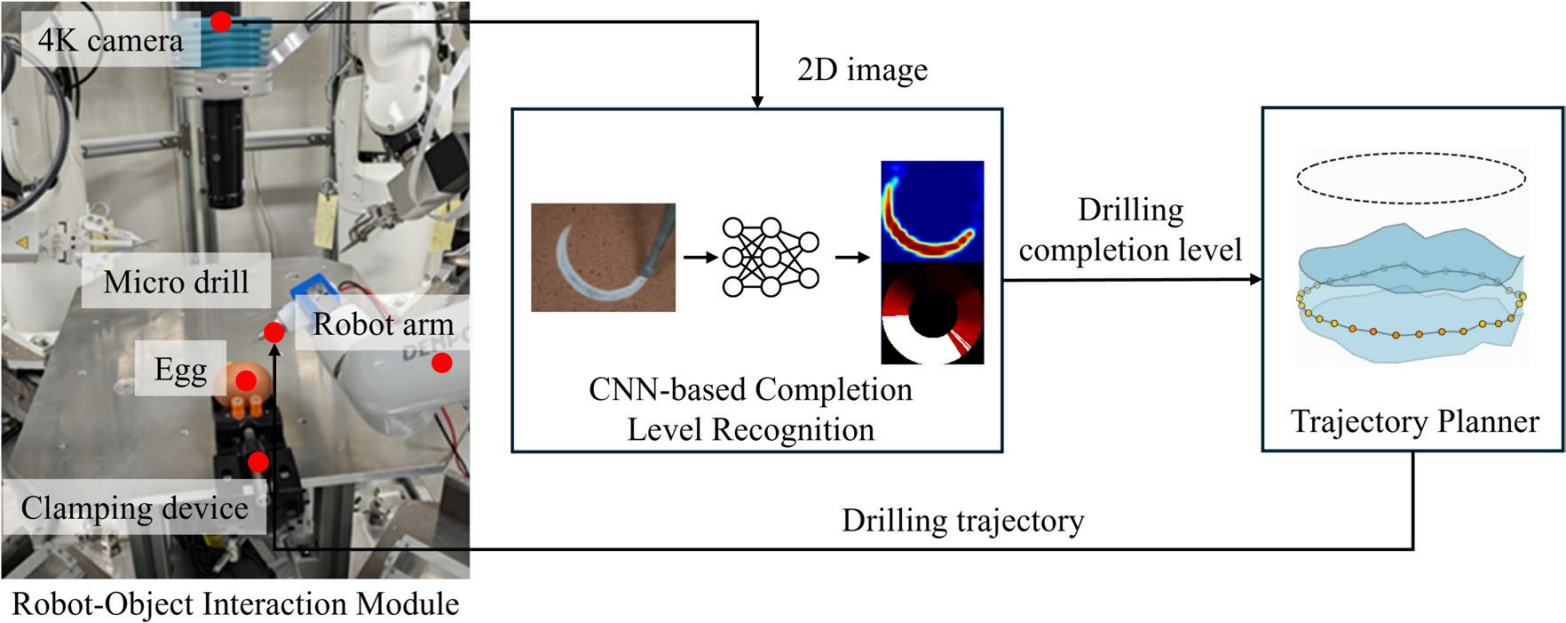
The autonomous robotic drilling system used for experiment.

### Setups

We apply the autonomous robotic drilling system developed in the previous study^15^ for the experiment. The system contains a Robot-Object Interaction Module, a CNN-based Completion Level Recognition and a Trajectory Planner, shown in Fig. 4.

The Robot-Object Interaction Module contains a 4K camera (STC-HD853HDMI, Omron-Sentech, Japan) with a distortionless macro lens of f=75 mm (VS-LDA75, VS Technology, Japan), a robot arm (CVR038, Densowave, Japan) holding a micro drill (MD1200, Braintree Scientific, USA), and a clamping device for fixing eggs. The 4K camera takes 2D images for eggshell drilling and send to CNN-based Completion Level Recognition in real time.

The CNN-based Completion Level Recognition generates a drilling progress bar based on the 2D image, and output the drilling completion level, which represents completion levels of measure points sampled along the trajectory. In the previous system, $f_R$ was applied as the CNN model that was trained on real dataset. In this experiment, in order to validate our proposed sim-to-real domain adaptation model, the CNN model $f_R$ was replaced by $f_T$.

The Trajectory Planner remains unchanged from the previous system, which can generate a continuous drilling trajectory based on the drilling completion level and send it the coordinate to robot controller as the target position of the micro drill.

### Software configuration

The experiments used a software implementation on a Ubuntu 20.04 x64 system. The robotic arm is controlled as described in the previous study^14^. ROS Noetic Ninjemys was used for the interprocess communication and CoppeliaSim for the simulations. Communication with the robot was enabled by the SmartArmStack. The dual quaternion algebra and robot kinematics were implemented using DQ Robotics^32^ with Python3. The force sensor was connected to a Raspberry Pi 4 Model B and the force data was read at 128 samples per second (SPS) using Python’s SMBus module and an I2C analogue input board.

### Metrics and baseline

Using $f_T$, 20 trials are conducted on eggs with varying shapes, sizes, and shell thickness. The success rate and average drilling time are used as evaluation metrics. A trial is considered successful if the shell can be cleanly removed with tweezers without damaging the membrane beneath. Failure is defined as membrane rupture, which typically occurs due to two reasons: network recognition errors and prolonged drilling duration, which will be discussed in detail later.

As a comparative baseline, 20 trials of experiments using $f_R$ was conducted in the previous study^15^, achieving a success rate of 80% and an average drilling time of 16.8 min. Among the 4 failure trials, 2 were caused by network recognition errors and the other 2 by prolonged drilling duration.

**Figure 5:**
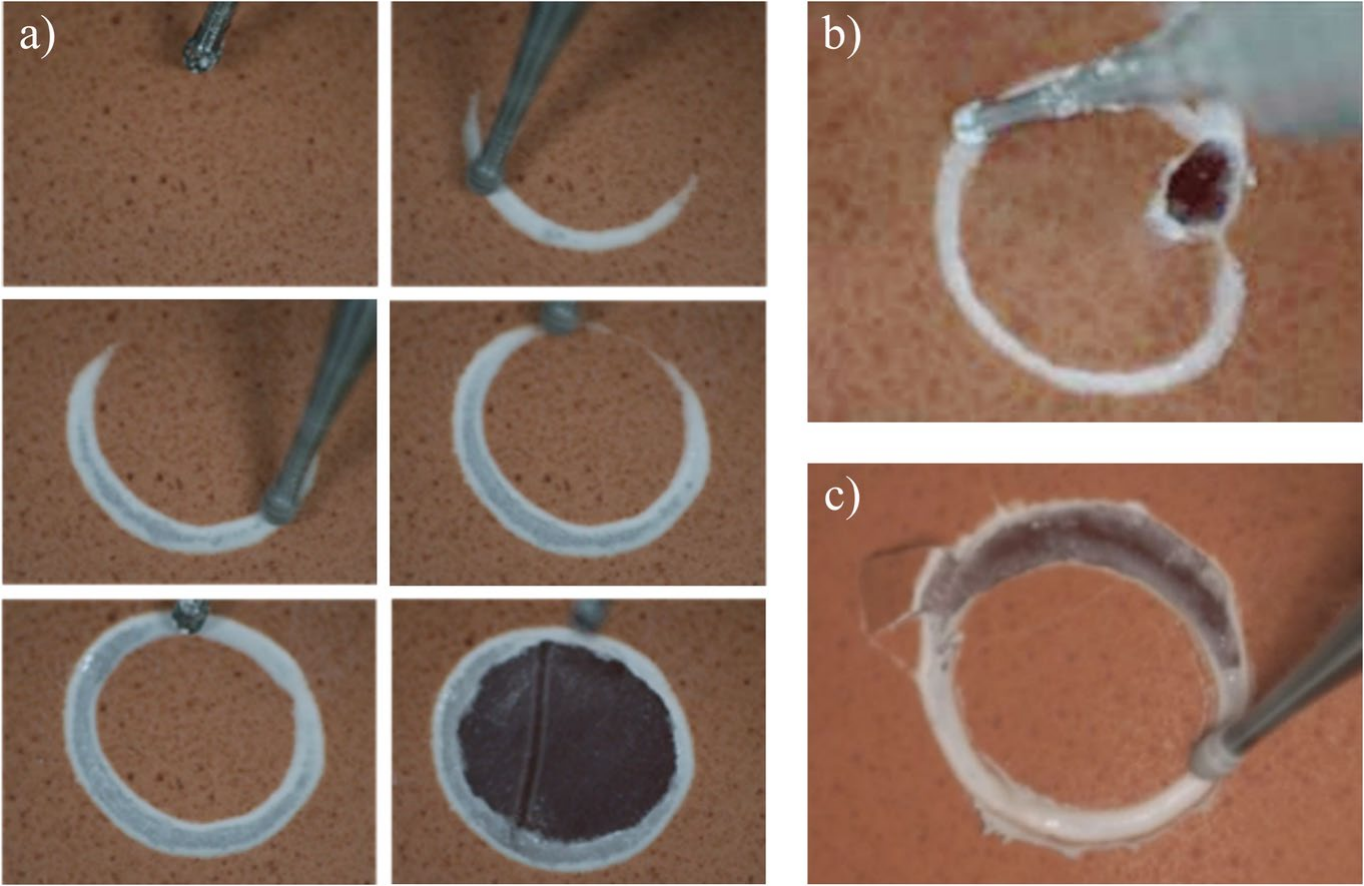
Example images of typical experimental results: (**a**) a series of screenshots of a successful trial, (**b**) failure due to recognition errors, (**c**) failure due to prolonged drilling duration.

### Results and analysis

In the experiment, 17 out of 20 trials were successful, resulting in a success rate of 85% and an average drilling time of 16.5 min. Figure 5a shows a successful example in which the drilling process automatically halts upon determining completion. In this trial, the circular patch is removed flawlessly without damaging the membrane.

Compared to baseline, $f_T$ shows a preliminary improvement in success rate (85% vs. 80%) while having little effect on drilling speed (16.5 min vs. 16.8 min) in eggshell drilling. The increase in success rate indicates that our proposed model $f_T$ can perform better than $f_R$. Although this may seem to contradict the comparison results in Table 1, which suggest that recognition on real images using $f_T$ is less accurate than $f_R$, this discrepancy can be explained by a crucial factor: the ground truths in Table 1 are based on manual annotation processed with Gaussian smoothing, which introduces annotation errors and smoothing-induced noise, making the ground truth inherently imprecise. These noisy labels limit the validity of the quantitative comparison and likely underestimate the true performance of $f_T$. We emphasize that the ultimate validation metric is not imperfectly annotated ground truth, but success of drill task. In this regard, the higher success rate of our sim-to-real model in the autonomous eggshell drilling provides preliminary evidence of its effectiveness and robustness. Regarding the almost unchanged drilling speed, our model was not designed to optimize this metric, so the results are consistent with expectations.

Moreover, experiment on $f_T$ still encountered 3 failure cases, where 1 was caused by network recognition errors (as shown in Fig. 5b) and 2 were due to prolonged drilling duration (an example shown in Fig. 5c). Compared to baseline, failures due to recognition errors were reduced, corresponding to the improved recognition accuracy discussed above. The only failure occurred when the drilling completion level had actually reached 100%, but the model incorrectly predicted it as incomplete, causing the drill to continue descending and puncture the membrane. We attribute this partially to the simulator, where the texture of the membrane exposed after penetrating the eggshell did not accurately reproduce the real color. This issue could be mitigated by further improving simulator fidelity. However, an autonomous drilling system relying solely on image feedback may have inherent systematic errors, and therefore, integrating other modalities may be necessary. On the other hand, failures due to prolonged drilling duration remained unchanged, which aligns with the previously discussed lack of improvement in drilling speed. These failures were attributed to the initial tilt of the egg, which caused the drill to contact different positions at significantly different times, thereby prolonging the drilling process and causing repeated friction on early-contacted areas of the membrane, leading to damage. This type of failure cannot be resolved by our current model and would require other approaches, such as 3D surface reconstruction, to improve drilling speed.

It is important to emphasize that while the experiment provided preliminary evidence of an improved success rate, which aligns with our theoretical analysis (as the model uses more precise synthetic ground truths), a limitation is that a Fisher’s exact test showed the result is not statistically significant ($p=0.6865$). This is likely due to the limited sample size of only 20 trials per group, a constraint imposed by sample availability. We plan to conduct more extensive trials in the future to provide a robust statistical confirmation.

## Discussion

Traditional automation methods for bone micro-drilling based on preoperative information rely on explicit knowledge of bone geometry and thickness, and require precise registration of these parameters to both the patient and the robot. However, their limited ability to respond to dynamic changes during drilling prevents them from achieving the required sub-millimeter accuracy. Learning-based strategies can overcome this limitation, but their deployment is often hindered by insufficient precision in collecting and manually annotating large training datasets and the substantial effort this process demands.

This study demonstrates that compared with training solely on real data, the proposed sim-to-real domain adaptation model provides significantly greater efficiency in dataset acquisition, and achieves comparable or even superior performance in autonomous eggshell drilling, thanks to the synthetic ground truths generated by simulator with higher accuracy.

Although our experiments were conducted on eggshell models, the underlying sim-to-real approach holds significant promise for automating micro-drilling in both rodents and human bone tissue. However, it is important to note that eggshells and real bone tissue differ in several key aspects, such as their mechanical properties, the presence of marrow and blood, and visual texture differences on their surfaces and cross-sections. These discrepancies could impact the model’s generalization ability and demand a higher degree of realism in the simulation environment. While image recognition alone was sufficient to verify task completion in the eggshell experiments, the presence of marrow and blood in rodents or human bone could degrade image recognition performance, thereby affecting the reliability of autonomous navigation. To address this challenge, future work should consider integrating force and other multimodal signals with visual information to achieve more robust control and recognition. Concurrently, the simulator can be expanded to not only generate visual features like texture differences but also provide force information and even simulate the effusion of blood. In principle, these functionalities are achievable with the micro-drilling simulator^17^, thereby providing more realistic and comprehensive data support for training.

Beyond experimental validation, these advances hold direct biomedical and clinical relevance, as they could ultimately enable autonomous assistance in real surgical scenarios, such as cranial window creation in rodents and cochlear implantation in humans, where precise and safe micro-drilling is critical. More generally, the proposed method could be adapted to handle a variety of complex and variable biomedical tissues beyond bone, provided that multimodal sensing and simulator fidelity are sufficiently enhanced. This highlights the broader potential of the sim-to-real pipeline to support automation in diverse biomedical and clinical procedures that require high-precision micro-drilling.

## Conclusion

This study presents a sim-to-real domain adaptation approach for recognizing completion levels in autonomous micro-drilling tasks, aiming to overcome the challenges of limited datasets and the inaccuracy and inefficiency of manual annotation in the training of supervised CNNs. By using a micro-drilling simulator to generate synthetic data and proposing a domain adaptation method to enable effective knowledge transfer from the synthetic to the real domain, the proposed method not only significantly accelerates dataset acquisition but also improves annotation accuracy. Autonomous eggshell drilling experiments demonstrated that the method improves the system’s success rate while maintaining a comparable average drilling time.

Future work will focus on improving the robustness of the model, expanding the diversity of the dataset, and extending the model to more complex biomedical and clinical scenarios.

## Additional information

**Correspondence** and requests for materials should be addressed to E.Z.

## Supplementary Information

Supplementary Information 1.

Supplementary Information 2.

## Data Availability

The synthetic and real-image datasets used for model training and evaluation during the current study are available from the corresponding author on reasonable request.

## Abbreviations

CNN: Convolutional neural network
CT: Computed tomography
OCT: Optical coherence tomography
DR: Domain randomization
DA: Domain adaptation
UDA: Unsupervised domain adaptation
GAN: Generative adversarial network
mAP: Mean average precision
mIoU: Mean intersection over union
MAPE: Mean absolute percentage error

## References

[1] Verillaud, B. et al. Endoscopic endonasal skull base surgery. *Eur. Annals Otorhinolaryngol. Head Neck Dis.* **129**, 190–196 (2012). 10.1016/j.anorl.2011.09.004 22321910

[2] Ogiwara, T., Goto, T., Hara, Y. & Hongo, K. Real-time navigation-guided drilling technique for skull base surgery in the middle and posterior fossae. *J. Neurol. Surg. Part B: Skull Base* **79**, S334–S339 (2018). 10.1055/s-0038-1667044 PMC6133694 30210987

[3] Balachandran, R. et al. Percutaneous cochlear implant drilling via customized frames: An in vitro study. *Otolaryngol. Head Neck Surg.* **142**, 421–426. 10.1016/j.otohns.2009.11.029 (2010). 20172392 10.1016/j.otohns.2009.11.029 PMC4425444

[4] Kanno, H., Handa, K., Murotani, M. & Ozawa, H. A novel intraoperative CT navigation system for spinal fusion surgery in lumbar degenerative disease: Accuracy and safety of pedicle screw placement. *J. Clin. Med.* **13**, 2105 (2024). 38610870 10.3390/jcm13072105 PMC11012415

[5] Gulner, B. R., Navabi, Z. S. & Kodandaramaiah, S. B. 3d morphometric analysis of mouse skulls using microcomputed tomography and computer vision. *bioRxiv* 2022–10 (2022).

[6] Bennett, C. et al. Shield: Skull-shaped hemispheric implants enabling large-scale electrophysiology datasets in the mouse brain. *Neuron* **112**, 2869–2885 (2024). 38996587 10.1016/j.neuron.2024.06.015

[7] Zorzos, A. N., Scholvin, J., Boyden, E. S. & Fonstad, C. G. Three-dimensional multiwaveguide probe array for light delivery to distributed brain circuits. *Opt. Lett.* **37**, 4841–4843 (2012). 23202064 10.1364/OL.37.004841 PMC3572236

[8] Shih, A. Y., Mateo, C., Drew, P. J., Tsai, P. S. & Kleinfeld, D. A polished and reinforced thinned-skull window for long-term imaging of the mouse brain. *J. Visualized Exp.: JoVE* 3742 (2012). 10.3791/3742 PMC3460568 22433225

[9] Silasi, G., Xiao, D., Vanni, M. P., Chen, A. C. & Murphy, T. H. Intact skull chronic windows for mesoscopic wide-field imaging in awake mice. *J. Neurosci. Methods* **267**, 141–149 (2016). 27102043 10.1016/j.jneumeth.2016.04.012 PMC5075450

[10] Shang, P. et al. Cutting-force modeling study on vibration-assisted micro-milling of bone materials. *Micromachines* **14**, 1422 (2023). 37512733 10.3390/mi14071422 PMC10384012

[11] Ghanbari, L. et al. Craniobot: A computer numerical controlled robot for cranial microsurgeries. *Sci. Rep.* **9**, 1023 (2019). 30705287 10.1038/s41598-018-37073-w PMC6355931

[12] Navabi, Z. S. *et al.* Computer vision–guided rapid and precise automated cranial microsurgeries in mice. *Sci. Adva.* **11**, eadt9693, 10.1126/sciadv.adt9693 (2025). https://www.science.org/doi/pdf/10.1126/sciadv.adt9693. 10.1126/sciadv.adt9693 PMC11980847 40203110

[13] Li, H. et al. Optical coherence tomography guided automatic robotic craniotomy surgery platform. *Biomed. Opt. Express* **16**, 778–789 (2025). 39958837 10.1364/BOE.549260 PMC11828463

[14] Marinho, M. M., Quiroz-Omaña, J. J. & Harada, K. A Multiarm robotic platform for scientific exploration: Its design, digital twins, and validation. *IEEE Robot. Autom. Mag.*10.1109/mra.2023.3336472 (2024).

[15] Zhao, E., Marinho, M. M. & Harada, K. Autonomous robotic drilling system for mice cranial window creation: An evaluation with an egg model. In *2023 IEEE/RSJ International Conference on Intelligent Robots and Systems (IROS)*, 4592–4599 (IEEE, 2023).

[16] Okuda, T., Kataoka, K. & Kato, A. Training in endoscopic endonasal transsphenoidal surgery using a skull model and eggs. *Acta Neurochir.* **152**, 1801–1804 (2010). 20700751 10.1007/s00701-010-0728-0

[17] Heredia Perez, S. A., Lok, T. L., Zhao, E. & Harada, K. A multimodal digital twin for autonomous micro-drilling in scientific exploration. *Int. J. Comput. Assist. Radiol. Surg.* 1–11 (2025). 10.1007/s11548-025-03465-3 PMC12518470 40569314

[18] Tremblay, J. *et al.* Training deep networks with synthetic data: Bridging the reality gap by domain randomization. In *Proceedings of the IEEE conference on computer vision and pattern recognition workshops*, 969–977 (2018).

[19] Loquercio, A. et al. Deep drone racing: From simulation to reality with domain randomization. *IEEE Trans. Robot.* **36**, 1–14 (2019).

[20] Tobin, J. *et al.* Domain randomization for transferring deep neural networks from simulation to the real world. In *2017 IEEE/RSJ international conference on intelligent robots and systems (IROS)*, 23–30 (IEEE, 2017).

[21] Tzeng, E., Hoffman, J., Saenko, K. & Darrell, T. Adversarial discriminative domain adaptation. In *Proceedings of the IEEE Conference on Computer Vision and Pattern Recognition (CVPR)* (2017).

[22] Ganin, Y. & Lempitsky, V. Unsupervised domain adaptation by backpropagation. In Bach, F. & Blei, D. (eds.) *Proceedings of the 32nd International Conference on Machine Learning*, vol. 37 of *Proceedings of Machine Learning Research*, 1180–1189 (PMLR, Lille, France, 2015).

[23] Ganin, Y. et al. Domain-adversarial training of neural networks. *J. Mach. Learn. Res.* **17**, 1–35 (2016).

[24] Chen, Y.-H. *et al.* No more discrimination: Cross city adaptation of road scene segmenters. In *Proceedings of the IEEE International Conference on Computer Vision (ICCV)* (2017).

[25] Hoffman, J. *et al.* Cycada: Cycle-consistent adversarial domain adaptation. In *International conference on machine learning*, 1989–1998 (Pmlr, 2018).

[26] Zhu, J.-Y., Park, T., Isola, P. & Efros, A. A. Unpaired image-to-image translation using cycle-consistent adversarial networks. In *Proceedings of the IEEE international conference on computer vision*, 2223–2232 (2017).

[27] He, K., Zhang, X., Ren, S. & Sun, J. Deep residual learning for image recognition. In *Proceedings of the IEEE conference on computer vision and pattern recognition*, 770–778 (2016).

[28] Tsai, Y.-H. *et al.* Learning to adapt structured output space for semantic segmentation. In *Proceedings of the IEEE conference on computer vision and pattern recognition*, 7472–7481 (2018).

[29] Li, Y., Shi, T., Zhang, Y. & Ma, J. Spgan-da: Semantic-preserved generative adversarial network for domain adaptive remote sensing image semantic segmentation. *IEEE Trans. Geosci. Remote Sens.* **61**, 1–17 (2023).

[30] Xu, T. et al. Fada: Feature aligned domain adaptive object detection in remote sensing imagery. *IEEE Trans. Geosci. Remote Sens.* **60**, 1–16 (2022).

[31] Lian, Q., Lv, F., Duan, L. & Gong, B. Constructing self-motivated pyramid curriculums for cross-domain semantic segmentation: A non-adversarial approach. In *Proceedings of the IEEE/CVF International Conference on Computer Vision*, 6758–6767 (2019).

[32] Adorno, B. V. & Marinho, M. M. DQ Robotics: a library for robot modeling and control. *IEEE Robot. Autom. Mag.(RAM)* **28**, 102–116, 10.1109/MRA.2020.2997920 (2021). Invited for presentation at IROS’21.

