# Supplementary material for: Sim-to-real domain adaptation based completion level recognition for autonomous micro-drilling in biomedical application
Source: Sci Rep. 2025 Nov 27;15:42417. doi: 10.1038/s41598-025-26600-1 (PMC12660929; doi:10.1038/s41598-025-26600-1)
Supplement: Supplementary file 1 — Supplementary Information 1. [file 41598_2025_26600_MOESM1_ESM.docx]

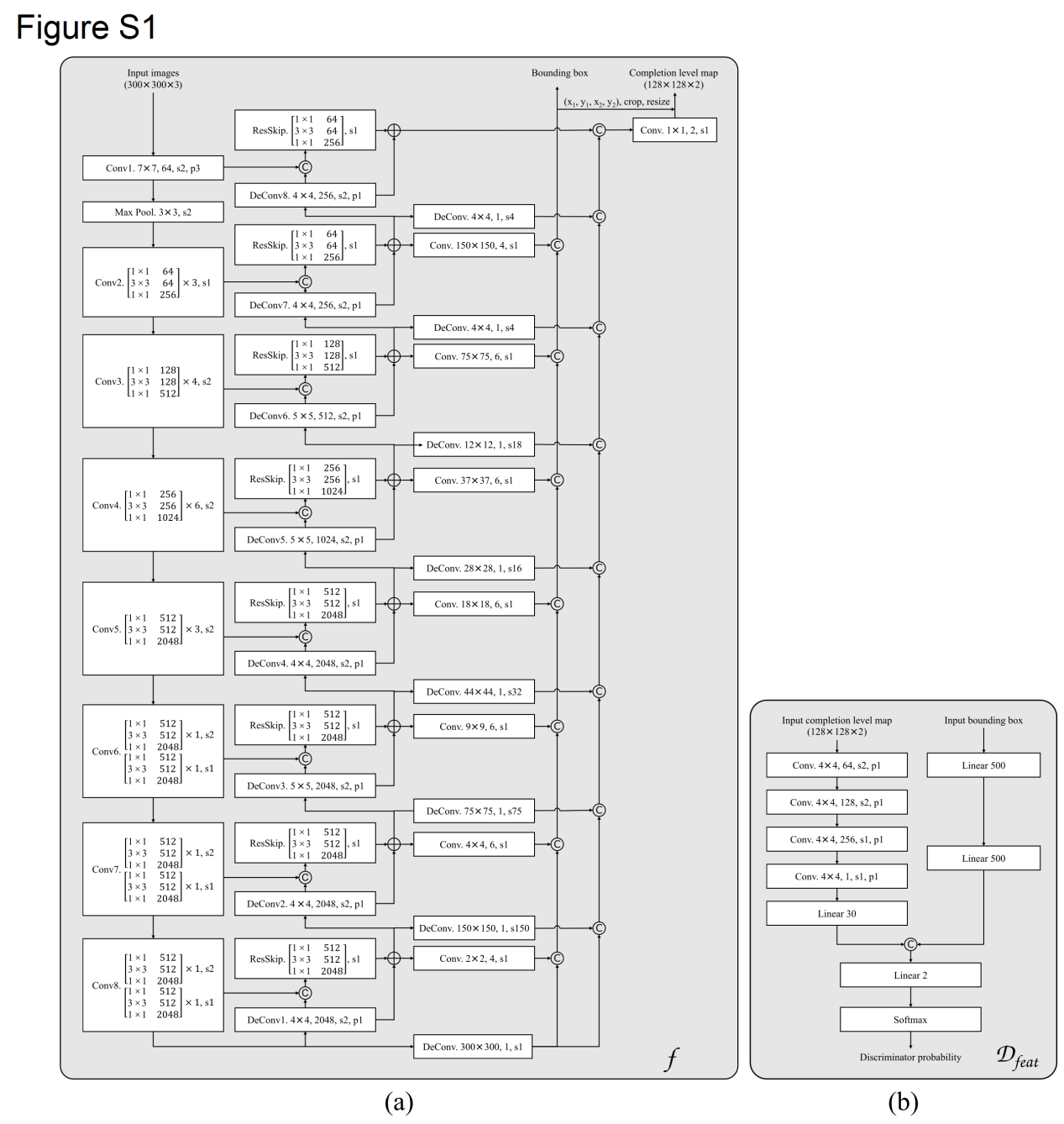


**Figure S1.** Network architectures of (a) task net (same for both $f_{S}$ and $f_{T}$) and (b) discriminator for feature level adaptation ($D_{feat}$). Note that the symbol © in the figure involves concatenating multiple tensors along a particular axis to create a single, unified tensor, while $\bigoplus$ refers to the summing of the values at corresponding positions of the input tensors.
