# Supplementary material for: Sim-to-real domain adaptation based completion level recognition for autonomous micro-drilling in biomedical application
Source: Sci Rep. 2025 Nov 27;15:42417. doi: 10.1038/s41598-025-26600-1 (PMC12660929; doi:10.1038/s41598-025-26600-1)
Supplement: Supplementary file 2 — Supplementary Information 2. [file 41598_2025_26600_MOESM2_ESM.docx]

**Table S2** Sensitivity analysis of task loss weight $\lambda_{task}$.

| $\lambda_{task}$ | mAP | mIoU ($\%$) | MAPE (%) |
| --- | --- | --- | --- |
| 0.7 | 68.1 | **81.0** | 22.8 |
| 0.8 | 68.4 | 80.9 | 22.8 |
| 0.9 | 69.1 | 80.3 | 22.7 |
| 1.0 | 69.3 | 80.9 | **22.8** |
| 1.1 | 69.5 | 80.4 | **22.8** |
| 1.2 | **70.1** | 79.9 | 23.1 |
| 1.3 | 68.5 | 80.0 | 23.0 |

**Table S3** Training configuration of task net for source domain.

| Details | Specification |
| --- | --- |
| Hardware | NVIDIA RTX A5000 GPU |
| Environment | Ubuntu 20.04, CUDA 11.3, Python 3.8, PyTorch 1.10.1 |
| Epoch | 6,000 |
| Batch size | 16 |
| Momentum | 0.9995 |
| Optimizer | Adam (Learning rate: 0.01, Beta1: 0.5, Beta2: 0.999) |

**Table S4** Training configuration of the domain adaptation model.

| Details | Specification |
| --- | --- |
| Hardware | NVIDIA RTX A5000 GPU |
| Environment | Ubuntu 20.04, CUDA 11.3, Python 3.8, PyTorch 1.10.1 |
| Epoch | 30 |
| Batch size | 1 |
| Momentum | 0.99 |
| Optimizer | Adam (Learning rate: 0.00001, Beta1: 0.5, Beta2: 0.9) |
